# Caffeic acid phenethyl ester protects against oxidative stress and dampens inflammation via heme oxygenase 1

**DOI:** 10.1038/s41368-018-0039-5

**Published:** 2019-02-20

**Authors:** Alexandra Stähli, Ceeneena Ubaidha Maheen, Franz Josef Strauss, Sigrun Eick, Anton Sculean, Reinhard Gruber

**Affiliations:** 1 Department of Oral Biology, School of Dentistry, Medical University of Vienna, Sensengasse 2a, Vienna, Austria; 2 Department of Periodontology, School of Dental Medicine, University of Bern, Freiburgstrasse 7, Bern, Switzerland; 3 Department of Conservative Dentistry, School of Dentistry, University of Chile, Sergio Livingstone 943, Santiago, Chile; 4 Austrian Cluster for Tissue Regeneration, Donaueschingenstrasse 13, Vienna, Austria

**Keywords:** Cell signalling, Genetics research

## Abstract

Periodontal disease is associated with chronic oxidative stress and inflammation. Caffeic acid phenethyl ester (CAPE), which is a potent inducer of heme oxygenase 1 (HO1), is a central active component of propolis, and the application of propolis improves periodontal status in diabetic patients. Here, primary murine macrophages were exposed to CAPE. Target gene expression was assessed by whole-genome microarray, RT-PCR and Western blotting. The antioxidative and anti-inflammatory activities of CAPE were examined by exposure of the cells to hydrogen peroxide, saliva and periodontal pathogens. The involvement of HO1 was investigated with the HO1 inhibitor tin protoporphyrin (SnPP) and knockout mice for Nrf2, which is a transcription factor for detoxifying enzymes. CAPE increased HO1 and other heat shock proteins in murine macrophages. A p38 MAPK inhibitor and Nrf2 knockout attenuated CAPE-induced HO1 expression in macrophages. CAPE exerted strong antioxidative activity. Additionally, CAPE reduced the inflammatory response to saliva and periodontal pathogens. Blocking HO1 decreased the antioxidative activity and attenuated the anti-inflammatory activity of CAPE. In conclusion, CAPE exerted its antioxidative effects through the Nrf2-mediated HO1 pathway and its anti-inflammatory effects through NF-κB inhibition. However, preclinical models evaluating the use of CAPE in periodontal inflammation are necessary in future studies.

## Healthy gums: a little help from honeybees

Propolis, also known as ‘honeybee glue,’ may protect teeth and gums against periodontal disease. In periodontal disease, chronic inflammation and oxidative damage harm gum tissue and lead to tooth loss; propolis has been shown to improve periodontal health for patients with diabetes. Bees make propolis by mixing beeswax, honey, plant resins and their own saliva, and use it to patch honeycomb and prevent growth of microbes in the hive. Reinhard Gruber of the Department of Oral Biology at the Medical University of Vienna and of the Department of Periodontology, University of Bern and co-workers investigated the effects of one of propolis’ active ingredients, caffeic acid phenethyl ester (CAPE), on oxidative stress and inflammation. They found that CAPE reduced oxidative damage and dampened inflammation; further investigation revealed the genetic basis of the beneficial effects, paving the way for future clinical studies. These results may help identify alternative treatments for periodontal disease.

## Introduction

Caffeic acid phenethyl ester (CAPE) is a central active component of propolis from honeybee hives. Propolis has multiple applications in dentistry. Systemic propolis improved the periodontal status in patients with type 2 diabetes,^1^ and local propolis application reduced plaque accumulation, gingival inflammation^2^ and oral mucositis during chemotherapy.^3^ In preclinical models, CAPE protected against ligature-induced periodontitis^4^ and systemic bone loss by cortisone,^5^ and supported bone defect healing.^6^ Therefore, CAPE is attracting attention in periodontology,^7,8^ and interest is growing in uncovering the beneficial effects of CAPE in periodontal therapy.

Periodontal disease is characterised by chronic inflammation^9^ and the concurrent challenge of oxidative stress,^10,11^ which together culminate in oral tissue destruction and ultimately tooth loss. Therefore, CAPE is of potential clinical interest. For example, CAPE prevents damage of cells exposed to hydrogen peroxide, including neurite PC12 cells,^12^ retinal 661W cells^13^ and umbilical vein endothelial cells.^14^ CAPE also exerts anti-inflammatory activity in gingival fibroblasts^7^ and macrophages^8^ exposed to endotoxins. Thus, CAPE supports the major defence mechanisms of cells challenged by oxidative stress and inflammation.

Nuclear factor erythroid 2-related factor 2 (Nrf2) is released from its suppressor Kelch-like ECH-associated protein 1 (Keap1) upon stimulation with CAPE.^15,16^ Nrf2 translocates into the nucleus and initiates the transcription of heme oxygenase 1 (HO1). HO1, which is linked to orthodontic tooth movement^17,18^ and inflammation in a periodontitis model,^10^ is a major target of CAPE. For instance, CAPE increases HO1 expression in gingival fibroblasts^7^ and macrophages.^8^ In turn, HO1 activates the cellular defence mechanisms against oxidative stress,^19^ including superoxide dismutase (SOD), catalase (CAT) and glutathione *S*-transferase (GST) expression.^20^ In support of this mechanism, Nrf2^21^ and HO1^22,23^ regulate SOD expression.

Tin protoporphyrin IX dichloride (SnPP) blocks HO1 activity, which allows insights into the role of HO1 in anti-inflammatory activity in macrophages^24^ and periodontal cells.^25^ Moreover, Nrf2 activation participates in the inhibitory effect of CAPE on nuclear factor-κappa-B (NF-κB) signalling.^26^ Independent of HO1, CAPE inhibits lipopolysaccharide (LPS)-induced interleukin (IL)-1β expression in RAW264.7 cells^8^ and inflammation in epithelial cells.^27^ However, CAPE requires HO1 to exert its anti-inflammatory activity, such as LPS-induced nitric oxide production, in RAW264.7 cells.^8^ Thus, the data are heterogeneous with respect to the ability of HO1 to mediate the anti-inflammatory activity of CAPE.

Mitogen-activated protein kinase (MAPK) may contribute to the CAPE-induced increase in HO1 expression. For example, the p38 MAPK inhibitor SB203580 attenuates the HO1 expression induced by CAPE.^28^ SB203580 also decreases HO1 expression in other in vitro models, including neuronal cells^29^ and RAW264.7 macrophages.^30–32^ Thus, the ability of CAPE to regulate HO1 and consequently to counteract oxidative stress and inflammation may involve p38 MAPK signalling.

The aim of the present study was to perform a genome-wide screening approach to evaluate CAPE-regulated genes and to use functional assays to better understand the role of HO1 in coordinating the cellular defence against oxidative stress and inflammation.

## Results

### CAPE increases heat shock proteins

**Fig. 1:**
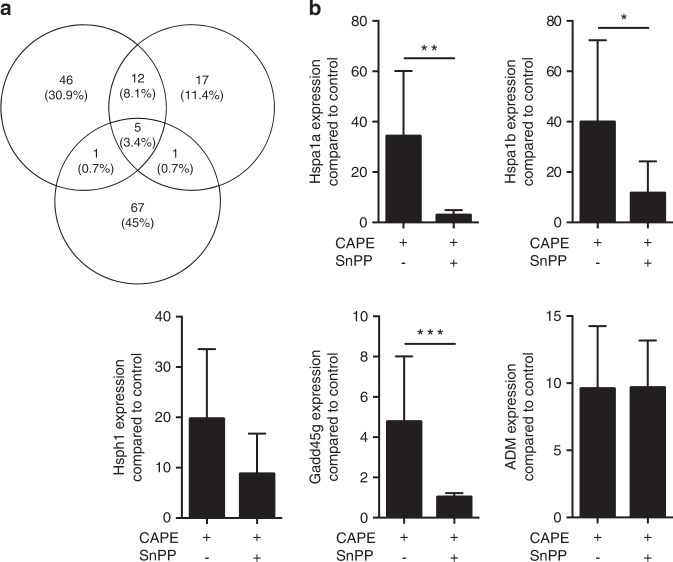
CAPE target genes assessed by microarray analysis and RT-PCR. Primary murine macrophages were incubated overnight with 10 µmol·L^−1^ CAPE. Microarrays of three independent experiments were analysed. Genes showing a more than 10-fold change were further analysed and compared among the three experiments. (**a**) The intersection of CAPE-induced genes between the experiments. RT-PCR was performed for Hspa1a, Hspa1b, Hsph1, Gadd45g and ADM (**b**), *n* = 3–5. The data represent the mean ± SD. *P < 0.05, **P < 0.01 and ***P < 0.001 in the two-tailed Mann-Whitney test

Based on a whole-genome microarray, treatment of three independent preparations of primary murine macrophages with CAPE caused expression changes of five characteristic stress-response genes more than 10-fold: Hspa1a (HSP70), Hspa1b, Hsph1 (HSP105, member of the HSP70 family), growth arrest and DNA-damage-inducible protein GADD45 gamma (Gadd45g)^33^ and adrenomedullin (ADM)^34^ (Fig. 1a). Increased Hspa1a, Hspa1b, Hsph1, Gadd45g and ADM expression was confirmed by reverse transcription-PCR (RT-PCR) analysis (Fig. 1b). Considering that heat shock proteins include HO1 (Hsp32), which is frequently co-expressed,^35^ and that CAPE is a known activator of HO1 expression in gingival fibroblasts^7^ and macrophages,^8^ we introduced SnPP, which is an inhibitor raised against HO1.^36^ SnPP almost abolished the CAPE-induced increase in Hsp and Gadd45g expression (Fig. 1c).

### CAPE increases HO1 expression

**Fig. 2:**
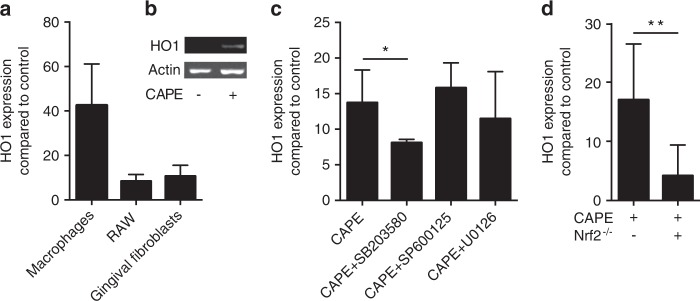
CAPE induces HO1 gene expression. Murine macrophages and RAW264.7 cells were stimulated overnight with 10 µmol·L^−1^ CAPE, and gingival fibroblasts were stimulated with 20 µmol·L^−1^ CAPE. HO1 gene expression was assessed (**a**), *n* = 3. HO1 protein expression was assessed by Western blotting in murine macrophages (**b**). Only relevant signals are shown. The full-length blots are presented in Supplementary Fig. 3. The impact of MAPK signalling was investigated using the respective inhibitors (SP600125 for JNK, SB203580 for p38 and U0126 for ERK) (**c**), *n* = 3. HO1 gene expression was further investigated in the Nrf2^−/−^ mice (**d**), *n* = 3. Data represent the mean ± SD. *P < 0.05 and **P < 0.01 in the two-tailed Mann-Whitney test

Next, we confirmed that CAPE was capable of increasing HO1 expression in murine macrophages and gingival fibroblasts. HO1 expression was considerably increased by CAPE in primary macrophages, RAW264.7 cells and gingival fibroblasts (Fig. 2a). HO1 was also increased at the protein level in macrophages as determined by Western blotting analysis (Fig. 2b). In macrophages, blocking p38 MAPK with SB203580 but not JNK and ERK attenuated the increase in HO1 expression (Fig. 2c). Moreover, cells from Nrf2-knockout mice displayed reduced HO1 expression in response to CAPE (Fig. 2d). Time-response experiments showed a peak in HO1 gene expression changes after 3–12 hours (Fig. S1).

### HO1 mediates the antioxidative activity of CAPE

**Fig. 3:**
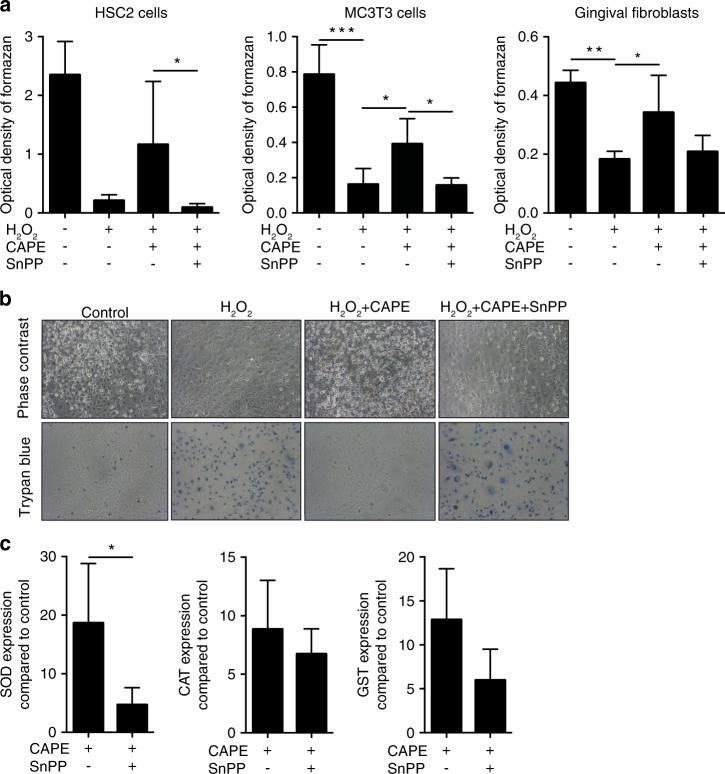
CAPE protects cells from oxidative stress. HSC-2, MC3T3-E1 cells and gingival fibroblasts were incubated with 1% H_2_O_2_ for 3 h with and without CAPE and the HO1 inhibitor SnPP. The MTT conversion assay showed that the presence of CAPE reduced the lethal effect of H_2_O_2_. Co-stimulation with SnPP reversed the rescue effects of CAPE (**a**), *n* = 3. The results were confirmed by phase contrast microscopy and trypan blue staining. Blue-stained cells represent dead cells (**b**), *n* = 3. Induction of the antioxidant enzymes superoxide dismutase (SOD), catalase (CAT) and glutathione *S*-transferase (GST) after CAPE stimulation was evaluated in murine macrophages (**c**), *n* = 3. The data represent the mean ± SD. *P < 0.05, **P < 0.01 and ***P < 0.001 in the Kruskal-Wallis test with Dunn’s multiple comparisons correction and the two-tailed Mann-Whitney test

To determine whether CAPE could support the ability of cells to cope with oxidative stress, various periodontal cell types were exposed to hydrogen peroxide. As predicted,^12–14^ hydrogen peroxide decreased the viability of HSC-2, MC3T3-E1 and gingival fibroblasts, as indicated by the reduced capacity to form formazan crystals (Fig. 3a) and reduced trypan blue staining of the HSC-2 cells (Fig. 3b). Blocking HO1 activity with SnPP reversed the positive effects of CAPE in the various cell types (Fig. 3a, b). Moreover, SnPP counteracted the increased SOD expression induced by CAPE (Fig. 3c). A trend towards lower CAT and GST expression by SnPP was also observed but failed to reach the level of significance (Fig. 3c).

### HO1 and inhibition of NF-κB subunit p65 control the anti-inflammatory activity of CAPE

**Fig. 4:**
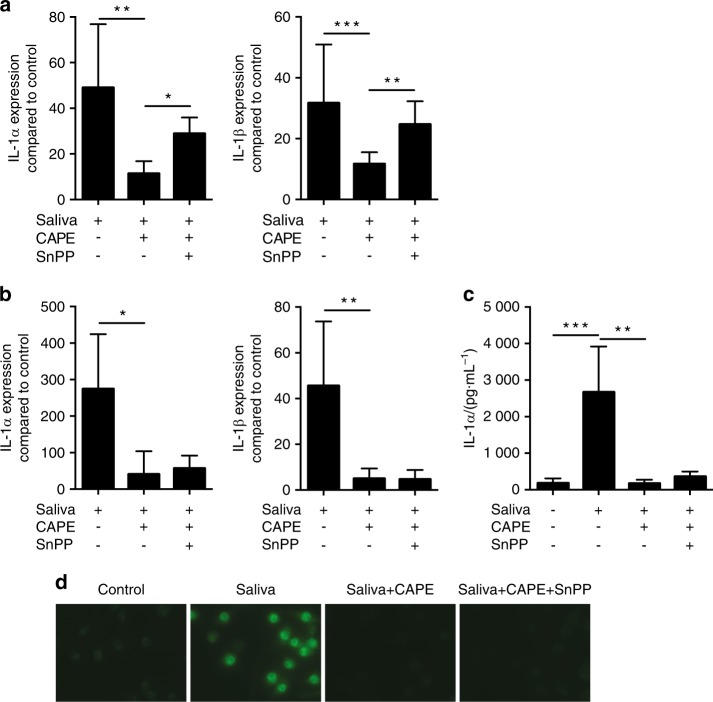
CAPE exerts anti-inflammatory effects. Cells were stimulated with 5% sterile filtered saliva and co-stimulated with CAPE and CAPE together with SnPP. IL-1α and IL-1β gene expression was assessed in both murine macrophages (**a**) and RAW264.7 cells (**b**), *n* = 3. The immunoassay for IL-1α confirmed the results at the protein level in RAW264.7 cells (**c**), *n* = 3. The data represent the mean ± SD. *P < 0.05, **P < 0.01 and ***P < 0.001 in the Kruskal-Wallis test with Dunn’s multiple comparisons correction. Immunofluorescence confirmed translocation of NF-κB p65 into the nucleus upon stimulation with saliva and its inhibition by CAPE (**d**)

**Fig. 5:**
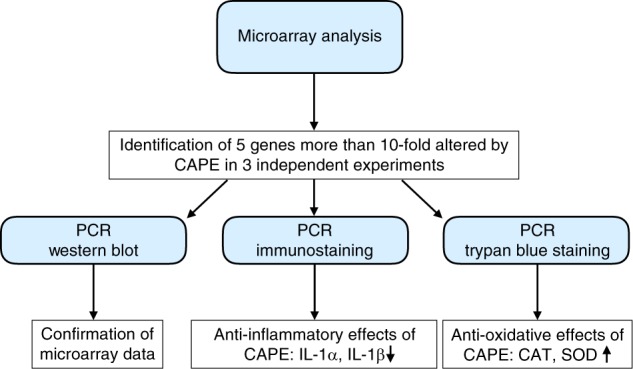
Flowchart of experiments. After identification of CAPE-induced genes by microarray analysis, the results were confirmed by PCR and Western blot. Functional analysis was performed using PCR, immunostaining or trypan blue staining

In support of previous data,^37^ primary macrophages showed a massive inflammatory response when exposed to human saliva (Fig. 4a). In the presence of CAPE, the strong proinflammatory response was almost abolished (Fig. 4a). Moreover, CAPE greatly suppressed the inflammatory response of macrophages exposed to supernatants of periodontal pathogens (e.g. *Porphyromonas gingivalis*, *Treponema denticola* and *Tannerella forsythia*) (Fig. S2). Next, we sought to investigate whether HO1 mediated the anti-inflammatory response of CAPE. SnPP reversed the anti-inflammatory effect of CAPE on saliva-induced IL-1α and IL-1β expression in primary macrophages but not in RAW264.7 cells (Fig. 4b, c). In support of previous findings,^8,38^ CAPE inhibited the translocation of p65 into the nucleus in RAW264.7 cells exposed to inflammatory clues. As expected, inhibition of p65 translocation by CAPE in RAW264.7 cells occurred independent of SnPP (Fig. 4d and Fig. 5).

## Discussion

Chronic inflammation in periodontitis is accompanied by oxidative stress, which together culminate in the catabolic events that ultimately lead to tissue destruction and tooth loss.^39,40^ One potential therapeutic strategy to regain periodontal health apart from scaling and root planing is to curtail inflammation and oxidative stress by local application of CAPE. Initial support for this hypothesis came from observations that propolis improved the periodontal status in type 2 diabetes patients^1^ and that CAPE protected against ligature-induced periodontitis in rats.^4^ CAPE also protects against other detrimental conditions, including peptidoglycan polysaccharide-induced colitis,^41^ Cr(VI)-induced brain toxicity,^42^ acetylsalicylic acid-induced lung damage,^43^ cyclosporine A-induced nephrotoxicity,^44^ cerulean-induced acute pancreatitis,^45^ cholestatic liver injury induced by bile duct ligation^46^ and acute myocardial ischaemia/reperfusion injury.^47^ Therefore, CAPE reduces detrimental effects related to inflammation and also exerts cytotoxic activity. Our intention was to extend the existing knowledge of how CAPE helps cells in the periodontium cope with inflammatory cues and hydrogen peroxide.

To screen for CAPE target genes, a microarray analysis was performed, which revealed a more than 10-fold increase in Hspa1a, Hspa1b, Hsph1, Gadd45g and ADM expression in murine macrophages. CAPE was reported to increase HspA5 expression in neuroblastoma cells^48^ and Hspa1a/Hspa1b expression in endothelial cells.^49^ With respect to possible relevance to periodontology, Hspa1a is upregulated in the periodontal ligament at the early stage of tooth movement in rats.^50^ Hydrogen sulphide-exposed oral keratinocyte stem cells express Gadd45g^51^ and ADM was detected in human gingival crevicular fluid.^52^ Moreover, we can reasonably suggest that Hsps, Gadd45g and ADM play a role in defence of the periodontium against oral pathogens, because recombinant Hsp70 protects cells from oxidative stress^53^ and reduces inflammation in sepsis models.^54^ Gadd45 proteins are implicated in cell cycle checkpoints, apoptosis and DNA repair.^55^ ADM not only regulates the vascular tonus but also has antioxidant and anti-inflammatory properties with therapeutic potential for myocardial infarction and inflammatory bowel diseases.^56^

Hspa1a, Hspa1b, Hsph1 and Gadd45g expression was reduced by blocking HO1 activity with SnPP. Surprisingly, HO1 did not reach the 10-fold expression level in the gene array that it achieved in the RT-PCR. As expected, HO1 expression was increased by CAPE in all cell types investigated, including primary macrophages, RAW264.7 macrophages and gingival fibroblasts. These findings support a role for CAPE as a potent agonist of HO1 expression in gingival fibroblasts^7^ and macrophages.^8^ However, we cannot state the percentage of HO1-positive cells, because no flow cytometry analysis was performed. To further explore the underlying signalling mechanism, the MAPKs were blocked. SB203580 attenuated the increased HO1 expression induced by CAPE. These findings are in support of the ability of p38 to regulate CAPE-induced HO1 expression in rat organotypic midbrain slice cultures.^28^ MAPK signalling has been implicated in Nrf2 induction. However, whether this effect occurs directly through phosphorylation of Nrf2 or through indirect and less specific mechanisms is unclear. Although inhibition of p38 abolished Nrf2 activation in glial cells,^57^ genetic deficiency or pharmacological inhibition of p38 increased HO1 expression through a Nrf2-dependent pathway.^58^ Considering the role of Nrf2 as an upstream regulator of HO1, cells from Nrf2-knockout mice displayed reduced HO1 expression in response to CAPE. These findings are in agreement with the release of Keap1 upon stimulation with CAPE,^15,16^ which allowed Nrf2 to initiate HO1 transcription.

In the present study, blocking HO1 with SnPP partially reversed the protective effects of CAPE on hydrogen peroxide damage similar to the other HO1 inducers quercetin^59^ and baicalein.^60^ In support of this functional assay, SnPP reduced CAPE-induced SOD expression. These findings were in line with reports showing that SnPP decreased SOD in smoke-induced emphysema rats.^61^ Additionally, a moderate decrease was observed in CAT, which is the enzyme that catalyses the decomposition of hydrogen peroxide to water and oxygen, and GST in this setting. Together, the data suggested that CAPE increased HO1 expression, which in turn drove SOD expression and probably that of other detoxifying enzymes to avoid hydrogen peroxide-induced cell death.

Here, SnPP increased the expression of inflammatory cytokines in primary macrophages, suggesting that the anti-inflammatory activity of CAPE required HO1 activity. However, SnPP did not modulate the anti-inflammatory effects of CAPE in RAW264.7 cells. In support of previous findings,^8,38^ CAPE inhibited translocation of p65 into the nucleus, thereby reducing the transmission of proinflammatory signals. Again, SnPP failed to reverse the effects of CAPE by blocking the translocation of p65 into the nucleus in RAW264.7 cells, suggesting that the anti-inflammatory activity of CAPE was independent of HO1 activity. Similar to our observations with primary macrophages, SnPP attenuated the inhibitory effects of flavonoids and hemin on LPS-induced inflammatory production in macrophages^24^ and sappanchalcone in LPS-stimulated human periodontal ligament cells.^25^ However, CAPE dampened inflammation in epithelial cells^27^ and LPS-induced IL-1β production in RAW264.7 cells^8^ independent of HO1 signalling. Therefore, HO1 is not necessarily involved in mediating the anti-inflammatory activity of CAPE in vitro. Nevertheless, our cumulative in vitro data suggest that CAPE-induced HO1 may be a central regulator that helps cells cope with oxidative stress and inflammation. Support for this hypothesis came from studies in which SnPP reversed the beneficial effects of baicalein and other HO1 inducers. For example, SnPP abolished the baicalein-, tetramethylpyrazine- and cepharanthine-mediated protection against ischaemia/reperfusion injury in various models.^22,62,63^ Future studies with CAPE should involve SnPP to reveal the role of HO1 in vivo.

The clinical relevance of the data presented here is based on revealing the molecular and cellular mechanisms of the beneficial effects of propolis on oral health.^1–3^ Its major bioactive component (CAPE) protects against ligature-induced periodontitis^4^ and systemic bone loss by cortisone^5^ and supports bone defect healing.^6^ Considering that periodontal disease is caused by chronic inflammation^9^ and oxidative stress^10,11^ and that CAPE suppresses inflammation and oxidative damage, our in vitro findings provide indirect support for HO1 as a target of periodontal therapy. Our data may also serve as a primer to extend this research to other fields in dentistry, including mucositis, peri-implantitis and pulpitis. However, care should be taken concerning the possible side effects of CAPE. For example, CAPE can have a strong antimitogenic activity in lung cancer, prostate cancer, melanoma,^64^ breast cancer^65^ and oral cancer.^66^ CAPE exerted cytotoxic effects on neck metastasis of gingiva carcinoma and tongue squamous cell carcinoma cells.^67^ Hence, safety and efficacy studies are required prior to clinical topical application of CAPE in the periodontium.

## Conclusion

Here we show that CAPE exerts its antioxidative effects through Nrf2-mediated HO1 expression and its anti-inflammatory activity through NF-κB inhibition.

## Materials and methods

### Murine bone marrow macrophages, human gingival fibroblasts and cell lines

Bone marrow cells were isolated from the femora and tibiae of BALB/c mice aged 6–8 weeks, seeded at a density of 1 × 10^6^ cells per cm^2^ into 12-well plates (CytoOne, Starlab GmbH, Hamburg, Germany) and grown for 7 days in Dulbecco’s modified medium (α-MEM) supplemented with 10% fetal bovine serum, 1% antibiotics (all from Invitrogen, Grand Island, NY, USA; growth medium) and 15% supernatant from L-929 cells (CCL-1™, American Type Culture Collection, Manassas, VA, USA).^68^ Osteoblastic MC3T3-E1 cells were originally obtained from Dr. Kumegawa (Josai Dental University, Sakado, Japan). RAW264.7 cells (ATCC^®^ TIB-71^™^) were obtained from the American Type Culture Collection. Human gingival fibroblasts were harvested from wisdom teeth extractions from patients who had given informed and written consent. Approval was obtained from the Ethics Committee of the Medical University of Vienna (EK NR 631/2007). All methods were performed in full accordance with the relevant guidelines and regulations. Tissue specimens were washed in phosphate-buffered saline (PBS) and culture medium for 2–3 min, cut into pieces 2 mm × 2 mm in size and placed into culture flasks. After 2–3 min, the flasks were flooded with culture medium (DMEM, pH 7.2) supplemented with 10% fetal calf serum and 1% antibiotics. Gingival fibroblasts that grew out from the explants were used for the experiments. Two strains of fibroblasts were established and were used for the experiments before passage 10. The oral squamous cell carcinoma cell line HSC-2 was obtained from the Japan Health Sciences Foundation (Health Science Research Resources Bank, JCRBD 6222). The cells were seeded at a concentration of 3 × 10^4^ cells per cm² into culture dishes 1 day prior to stimulation. The CAPE concentration was evaluated by 3-[4,5-dimethythiazol-2-yl]−2,5-diphenyltetrazolium bromide (MTT) assay (data not shown) and was in line with the concentrations reported in the literature. Cells were exposed to CAPE (Sigma, St. Louis, MO, USA) at a 10-µmol·L^−1^ concentration in serum-free medium either alone or in the presence of its inhibitor tin protoporphyrin IX dichloride (SnPP; Sigma) at a concentration of 10 µmol·L^−1^ for 12 hours. To block the main MAPK signalling pathways, the pharmacologic inhibitors SP600125, SB203580 and U0126 were used, all at 10-µmol·L^−1^ concentrations (Santa Cruz Biotechnology, SCBT; Santa Cruz, CA, USA).

### Cell viability

For the viability experiments, the cells were incubated with CAPE with or without SnPP for 30 min prior to exposure to 1% H_2_O_2_. After 3 h, the cell viability assay and trypan blue staining were performed. For cell viability, MTT (Sigma) solution at a final concentration of 0.5 mg·mL^−1^ was added to each well of a microtiter plate (CytoOne) and incubated for 2 h at 37 °C. Then, the medium was removed, and the formazan crystals were solubilized with dimethyl sulphoxide. The optical density was measured at 570 nm. The data are expressed as percentages of the optical density in the treatment groups normalised to the unstimulated control values. Moreover, 50 µL of 0.4% trypan blue (Sigma) was added to each well and incubated for 5 min at room temperature. Then, the trypan blue solution was discarded, and the cells were examined by microscopy and photographed.

### Cell inflammatory response

For the inflammation experiments, the cells were incubated with CAPE with or without SnPP prior to exposure to 1% sterile saliva. After 24 h, the supernatant was harvested, and RNA was isolated. Whole human saliva was collected from two authors (R.G. and A.S.), who were non-smokers and gave their informed consent. Saliva flow was stimulated by chewing paraffin wax (Ivoclar Vivadent AG, Schaan, Liechtenstein) without eating and drinking for 1 h prior to collection. Immediately after collection, the saliva was centrifuged at 4 000 × *g* for 5 min. The saliva supernatant was passed through a filter with a 0.2-µm pore diameter (Diafil PS, Graphic Controls/DIA-Nielsen GmbH & Co. KG, Düren, Germany).

### Whole-genome gene array

Total RNA was harvested from primary murine macrophages with the RNA Isolation Kit (Extractme, BLIRT S.A., Gdańsk, Poland). The RNA quality was determined using the Agilent 2100 Bioanalyzer (Agilent Technologies, Santa Clara, CA, USA). A total of 100 ng of total RNA was amplified and labelled using the Genechip^®^ WT PLUS Reagent Kit (Catalogue Number 902281, Affymetrix, Santa Clara, CA, USA). Labelled RNA samples were hybridised onto the Affymetrix GeneChip^®^ Mouse Gene 2.1 ST Array. The array plate was washed, stained and scanned using the Affymetrix Gene Titan according to the Gene Titan^®^ Instrument User Guide for expression array plates. Genes with at least 10-fold regulation in three independent experiments were selected with Venny (http://bioinfogp.cnb.csic.es/tools/venny/).

### RT-PCR and immunoassay

RT was performed with the SensiFAST™ cDNA Synthesis Kit (Bioline Reagents Ltd., London, UK). The RT-PCR was performed with the SensiFAST™ SYBR^®^ Kit following the manufacturer’s instructions (Bioline). Amplification was performed with the StepOnePlus Real-Time PCR System (Applied Biosystems, Life Technologies, Carlsbad, CA, USA). The primer sequences are given in Table 1. Relative gene expression was calculated with the delta delta CT method. The reactions were run in duplicate. The IL-1α in the supernatant was analysed using an immunoassay kit according to the manufacturer’s instructions (R&D Systems, Minneapolis, MN, USA).

**Table 1:** Primer sequences

Primer	Sequence_F	Sequence_R
mHO1	CAATGTGGGCTTCTC	TTTTGGTGAGGGAAC
mHspa1a	GGCCAGGGCTGGATTACT	GCAACCACCATGCAAGATTA
mHspa1b	GAAGACATATAGTCTAGCTGCCCAGt	CCAAGACGTTTGTTTAAGACACTTT
mHsph1	TGTTTTGCATTTTGCTCTCTTCA	AAAGCCCTTATGCAAATCAGAC
mGadd45g	GAATAACTTGCTGTTCGTGGA	AAGTTCGTGCAGTGCTTTCC
mADM	GGACACTGCAGGGCCAGAT	GTAGTTCCCTCTTCCCACGACTTA
mIL1a	TTGGTTAAATGACCTGCA	GAGCGCTCACGAACAGT
mIL1b	AAGGGCTGCTTCCAAACCTTTGAC	ATACTGCCTGCCTGAAGCTCTTGT
mIL6	GCTACCAAACTGGATATAATCAGGA	CCAGGTAGCTATGGTACTCCAGAA
mSOD	CTCTTGGGAGAGCCTGACA	GCCAGTAGCAAGCCGTAGAA
mCAT	CCTTCAAGTTGGTTAATGCAGA	CAAGTTTTTGATGCCCTGGT
mGST	GGCAGAATGGAGTGCATCA	TCCAAATCTTCCGGACTCTG
mGAPDH	AACTTTGGCATTGTG	GGATGCAGGGATGAT
mbactin	CTAAGGCCAACCGTGAAAAG	ACCAGAGGCATACAGGGACA
hHO1	ACATCTATGTGGCCCTGGAG	GTTGAGCAGGAACGCAGTCT
hGAPDH	AAGCCACATCGCTCAGACAC	GCCCAATACGACCAAATCC
hactin	CCAACCGCGAGAAGATGA	CCAGAGGCGTACAGGGATAG

### Western blotting

Macrophages were serum-starved overnight and then treated overnight with CAPE as indicated. Cell extracts containing SDS buffer and protease inhibitors (PhosSTOP with cOmplete; Sigma, St. Louis, MO, USA) were separated by SDS-polyacrylamide gel electrophoresis and transferred onto nitrocellulose membranes (Whatman, GE Healthcare, General Electric Company, Fairfield, CT, USA). The membranes were blocked, and binding of the primary antibody (HO1 polyclonal antibody, Enzo Life Sciences, Farmingdale, NY, USA) was detected with an appropriate secondary antibody directly labelled with near-infrared dyes (LI-COR Biosciences, Lincoln, NE, USA) and visualised with an appropriate imaging system (LI-COR). The acquired images were not processed.

### Bacterial cultures

Bacterial cultures of *P. gingivalis*, *T. denticola* and *T. forsythia* were grown in Schaedler broth (Oxoid Basingstoke, GB) supplemented with 0.25 mg·L^−1^ of vitamin K (and 10 mg of *N*-acetyl muramic acid for *T. forsythia*) and modified mycoplasma broth (BD, Franklin Lake, NJ, USA) supplemented with 1 mg·mL^−1^ of glucose, 400 mg· mL^−1^ of niacinamide, 150 mg·mL^−1^ of spermine tetrahydrochloride, 20 mg·mL^−1^ of Na isobutyrate enriched with 1 g·mL^−1^ of cysteine and 5 mg·mL^−1^of cocarboxylase. Forty-eight-hour broth cultures of the bacteria were centrifuged. The resulting supernatants were filtered through 400-µm membranes, and the sediments were exposed to intensive ultrasonication for 10 min.

### Immunostaining

Immunofluorescent analysis of NF-κB p65 was performed in RAW264.7 cells plated onto Millicell^®^ EZ slides (Merck KGaA, Darmstadt, Germany) and treated with 10 μmol·L^−1^ CAPE for 30 min prior to exposure to 1% saliva. The cells were fixed in paraformaldehyde and blocked with 1% bovine serum albumin and 0.3% Triton in PBS at room temperature for 1 h. Then, the cells were incubated with a NF-κB p65 primary antibody (1:100; Cell Signaling Technology, USA). An Alexa 488 secondary antibody (1:200, Santa Cruz Biotechnology, USA) was applied for 1 h. The cells were washed, the nuclei were stained with 4′,6-diamidino-2-phenylindole (100 ng·mL^−1^) and the cells were mounted onto glass slides. Fluorescent images were captured at ×100 in oil immersion using a Zeiss Axiovert 200M fluorescence microscope.

### Statistical analysis

The primary culture experiments were performed at least three times. All experiments were repeated three to five times. The bars show the means and standard deviations of cumulative data from all experiments. The statistical analysis was based on the Mann-Whitney *U* test and Kruskal-Wallis test with Dunn’s multiple comparisons correction. The analyses were performed using Prism v7 (GraphPad Software, La Jolia, CA, USA). Significance was set at *P* < 0.05.

## Electronic supplementary material

Supplement 4

Supplement 1

Supplement 2

Supplement 3

## Data Availability

The data sets generated during and/or analysed during the current study are available from the corresponding author upon reasonable request.
